# Systematic Review and Meta-Analysis on the Prevalence and Antibiotic Susceptibility Pattern in *Pseudomonas aeruginosa *Isolated from Cystic Fibrosis Patients

**DOI:** 10.5152/eurasianjmed.2024.23302

**Published:** 2024-10-01

**Authors:** Ali Rastegar-Kashkouli, Azad Khaledi, Farzaneh Moammer, Mohammad Ghenaatpisheh Sanani, Mohammad Mahdi Heidari, Aynaz Foroughi Eghbal

**Affiliations:** 1 Isfahan University of Medical Sciences School of Medicine, Isfahan, Iran; 2 Infectious Diseases Reaserch Center, Kashan University of Medical Sciences, Kashan, Iran; 3 Student Research Committee, Guilan University of Medical Sciences School of Medicine, Guilan, Iran; 4 Kowsar Hospital Research Committee, Fars Heart Foundation, Shiraz, Iran; 5 Department of Pediatrics, Kashan University of Medical Sciences Faculty of Medicine, Kashan, Iran; 6 Urmia University of Medical Sciences School of Medicine, Urmia, Iran

**Keywords:** Antibiotic resistance, colistin, *Pseudomonas aeruginosa*, cystic fibrosis, Middle East

## Abstract

This study aimed to conduct a retrospective Middle East systematic review and meta-analysis on the prevalence and antibiotic susceptibility pattern for this microorganism isolated from cystic fibrosis patients. We searched MEDLINE, the Cochrane Library, SCOPUS, and Web of Science (ISI) to identify studies that reported the prevalence of *Pseudomonas aeruginosa *isolated from cystic fibrosis (CF) patients, and antibiotic resistance patterns. To assess the quality of publications was used of a checklist provided by the Joanna Briggs Institute. Finally, the data was analyzed by comprehensive meta-analysis software. The studied populations comprised children and young, and adult CF patients. Patients were aged between 3 months-65 years. A higher percentage of CF patients were males. *Pseudomonas aeruginosa *frequency varied between 5.9 and 76.2% in the studies included. The combined prevalence of *P. aeruginosa *was reported 34.3%. The lowest level resistance of *P. aeruginosa *was toward colistin (0%-13.3%) and ticarcillin (3.9%-24%). Our study showed the prevalence of *P. aeruginosa *and antibiotic resistance are almost high, while colistin and ticarcillin are the best antibiotics to decrease postantibiotic effects (PAEs) in CF patients from the Middle East. Therefore, physicians should pay more attention to therapeutic protocols to prevent further resistance.

**Main Points**

- Pseudomonas aeruginosa frequency varied between 5.9 and 76.2% in this review.
- The combined prevalence of *P. aeruginosa* was reported 34.3%.
- The lowest level resistance of *P. aeruginosa* was toward colistin.

## Introduction

Cystic fibrosis (CF), an autosomal recessive progressive condition triggered by a mutation in the gene encoding the CF transmembrane (CFTR), is characterized as chronic obstructive pulmonary disease, exocrine pancreatic insufficiency, and elevated sodium and chloride concentrations in sweat.^1^ Electrolyte imbalance on both sides of the membrane decreases the volume of water in the secretions of the apocrine glands, which are very viscous and result in the narrowing of the duct, blockage, and subsequent dissolution of these glands. So, the moisture on the surface of the main respiratory tract is greatly decreased, and the condition is primed for the invasion of pathogens.^2^

Cystic fibrosis is more common in white people in Europe, North America, and Australia, although the disease affects people of all races.^3^ In the United States, for example, about 30 000 individuals are estimated to have CF, and more than 1000 new cases are found each year.^4^ This disease is very rare in Arab cultures, but it is equally widespread in white populations.^5^ Many CF patients will begin to experience chronic hypoxic and hypercapnic respiratory failure, as well as pulmonary exacerbations, atypical respiratory pathogen acquisition, pneumothorax, hemoptysis, pulmonary hypertension, and pulmonary hypertension development.^6^ The presence of elevated sweat chloride confirms the diagnosis of CF. The majority of CF cases are discovered by newborn screening.^7^

Many bacterial species have been linked to CF respiratory tract infections, including *Pseudomonas aeruginosa*, *Stenotrophomonas*, *Maltophilia*, *Staphylococcus aureus*, and *Haemophilus influenza*.^7^ *Pseudomonas aeruginosa* aggravates lung function loss and is linked to high morbidity and mortality in CF patients.^8^ *P. aeruginosa* has been found in 60.9% of CF patients with CFTR mutations’ lower respiratory samples,^9^ and this mutation is one of the most frequent among Arabs in the Gulf region who belong to a large Arab tribe.^10^ The prevalence of *P. aeruginosa* increases with age, making it the most common pathogen in the elderly.^11-16^ Therapeutic advances, such as a greater emphasis on improving diet with multivitamins and pancreatic enzymes, airway clearing treatments, antibiotics, and highly efficient CFTR modulators, have resulted in improved clinical conditions and longevity for CF patients.^17^ Many times in the past, the life expectancy of CF patients has often improved due in part to appropriate antibiotic therapy in the procedure.^18^ *P. aeruginosa* is still not eradicated in such patients, and the emergence of different bacterial morphotypes during chronic infection has been identified globally.^19,20^

This microorganism has acquired an intrinsic antibiotic resistance which severely limits effective therapeutic options, especially in patients with CF and who have been thermally burnt.^21^ According to some reports, extended spectrum beta-lactamases (ESBLs) and metallo-β-lactamases (MBLs) are becoming more common among *P. aeruginosa* in Middle Eastern Arab countries, which may result in a longer hospital stay, higher costs, and a fatal outcome for patients.^22-24^ The presence of an alginate-containing matrix in mucoid strains causes them to be more resistant to antibiotics. Eradication methods not only harm non-mucoid strains but can also hinder disease growth.^25^ Because of the repeated and sometimes extended courses of nasal, intravenous, and aerosolized antibiotics used to treat chronic lung disease, CF patients are at extremely high risk of contracting infections with multidrug-resistant (MDR) pathogens, especially *P. aeruginosa*. Furthermore, the Arabic-speaking population in the Middle East has a high prevalence of CFTR gene mutation.^26^ In the management of lung infection resulting from *P. aeruginosa*, inhaled antibiotics such as azithromycin, aztreonam, and tobramycin are used as maintenance drugs.^27^ According to guidelines, continuous use of these antibiotics improves pulmonary function in patients with CF.^28^ They also reduce exacerbations in chronic infection caused by *P. aeruginosa.* ^28^

Since the extent of antibiotic resistance of *P. aeruginosa* recovered from CF patients in the Middle East has not been comprehensively addressed, we decided to conduct a retrospective Middle East systematic review about resistance to key antibiotics used in the treatment of *P. aeruginosa* infection and the prevalence of *P. aeruginosa* isolated from CF patients, too.

## Material and Methods

### Search Strategy

A systematic literature search of English-language studies was conducted from 2000-January 1, 2024, through Medline, Web of Science, Scopus, Google Scholar, and Cochrane using the following search terms: (“Cystic Fibrosis” OR “Mucoviscidosis” OR “Pulmonary Cystic Fibrosis” OR “CF”) AND (“*Pseudomonas aeruginosa”* OR “*P. aeruginosa”) *AND (*“*Middle East”)*, *AND (“Drug Resistances” OR “Antimicrobial Drug Resistance” OR “Antibiotic Resistance”). The reference lists of the included studies were surveyed for further eligible articles. The process was conducted by 2 independent assessors. Also, disagreements between investigators were resolved by agreement.

### Inclusion and Exclusion Criteria

The articles that used standard molecular and phenotypic methods for isolation of *P. aeruginosa*; and studies that reported the prevalence of *P. aeruginosa* isolated from CF patients were included. In contrast, studies that did not report the prevalence of *P. aeruginosa *among CF patients, systematic reviews, meta-analyses, narrative reviews, editorials, case reports, abstracts, and duplicate articles were excluded.

### Quality Assessment

The quality of studies included was assessed using a checklist provided by the Joanna Briggs Institute (JBI).^29^ In total, title and abstract, introduction, methods, results, and discussion items were assessed through questions designed by JBI, and a score was allocated to each section.

### Data Extraction

First author, time of the study, publication time, type of study, location of the study, genus, age, sample size, source of samples, the prevalence of *P. aeruginosa, *MDR isolates, mucoid, non-mucoid, and patient’s condition were extracted from studies included in the present systematic review.

## Results

### Study Characteristics

**Figure 1.**
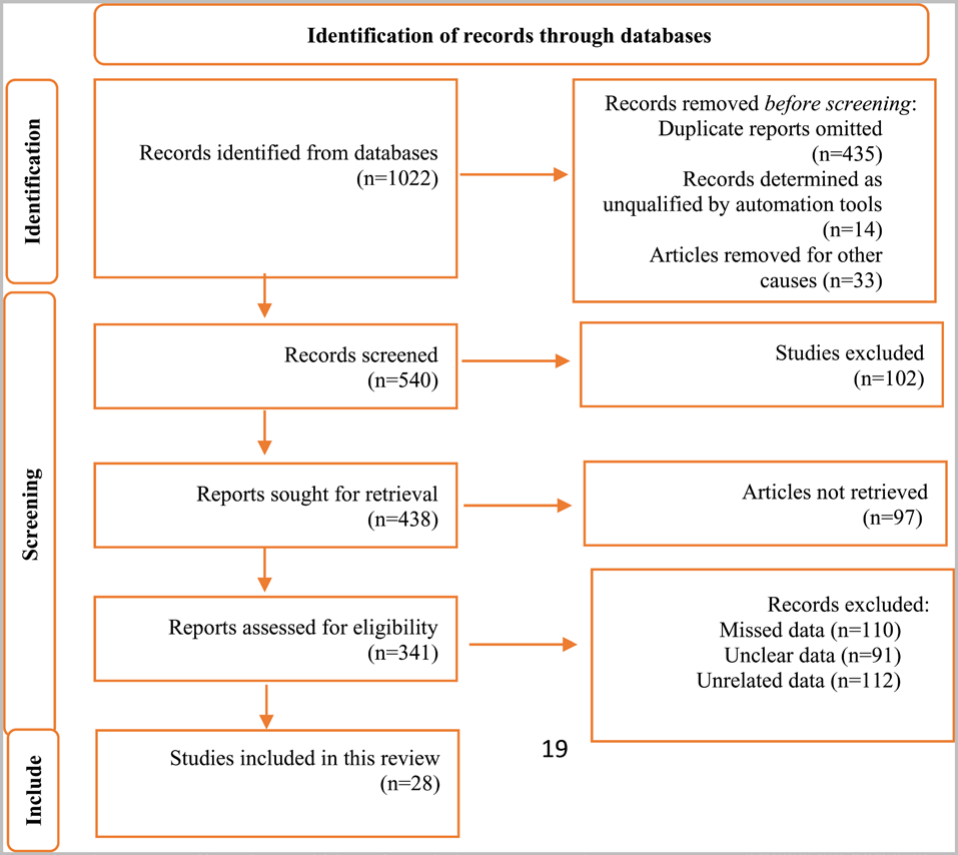
Flow diagram for included searches of databases and registers.

Of a total of 1022 studies recognized by the searches, 482 records were removed before screening. Then, 540 articles were screened, and 102 records were excluded from screening with reasons. Four hundred thirty-eight reports were sought for retrieval, but 97 reports were not retrieved. Three hundred forty-one reports were assessed for eligibility, and 313 studies were excluded with reasons. Finally, 28 studies were included in the qualitative systematic review, as shown in Table 1 and Figure 1.

This review was conducted to cover the studies carried out all over the Middle East countries. The distribution of 28 studies included was as follows: 10 from Türkiye, 9 from Iran, Jordan, Qatar, and Iraq each had 2 studies, also, Saudi Arabia, Egypt, and Syria each had 1 study while according to quality assessment, no study from Bahrain, Oman, Cyprus, Yemen, United Arab Emirates, Palestine, Lebanon, and Kuwait met the eligibility to be included. The sample size ranged from 16 to 1767. Most studies were cross-sectional (n = 19), cohort (n = 3), longitudinal (n = 1), and 5 of them did not report the type of study. In most studies, the sources of samples were sputum, followed by throat swabs, bronchoalveolar lavage, and different samples (Table 1). Also, 17 studies met the eligibility criteria for inclusion in the antibiotic resistance section.

### Characteristics of cystic fibrosis patients

The studied populations comprised children, young, and adult CF patients. Patients were aged between 3 months and 65 years. According to the data extracted in Table 1, some studies reported a high percentage of females while other studies reported a high percentage of males with CF, but in total, a higher percentage of CF patients were male. Some studies reported the condition of patients as chronic, while some studies did not report this.

### *Pseudomonas aeruginosa* Isolates

**Figure 2.**
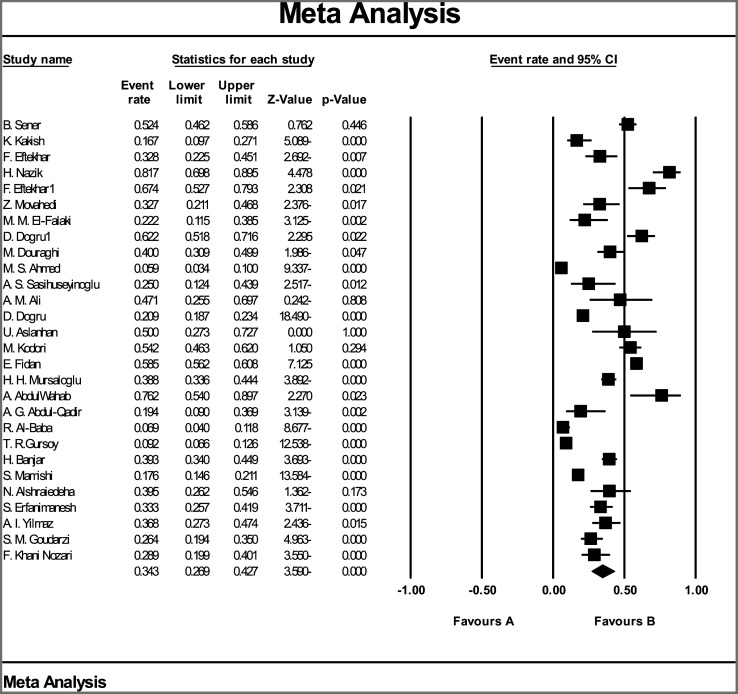
Forest plot of the meta-analysis of the prevalence of *Pseudomonas aeruginosa* isolated from patients suffering from cystic fibrosis.

As abstracted in Table 1,* P. aeruginosa* frequency varied between 5.9% and 76.2% in studies included. Also, data from Figure 2 showed the combined prevalence of *P. aeruginosa *34.3% (95% CI, 26.6-42.7). Prevalence of MDR* P. aeruginosa* isolates was reported in 3 studies; M.S. Ahmed and et al. (8.1%),^30^ and Eftekhar et al. (9.5%), and Soroor Erfanimanesh et al (25.5%).^31^

### Heterogeneity and Publication Bias

**Figure 3.**
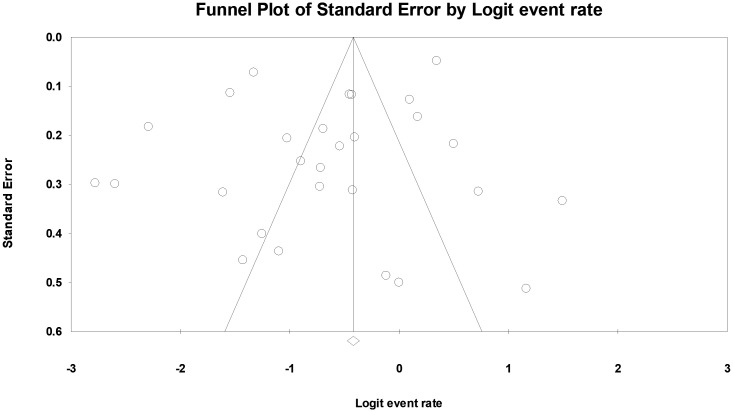
Funnel plot of the meta-analysis of the prevalence of *Pseudomonas Aeruginosa* isolated from patients suffering from cystic fibrosis.

Data obtained in this meta-analysis showed heterogeneity (*Q* = 875.7 and *Z *= 3.6, and *I* ^2^ = 97). A glance at the funnel plot (Figure 3) indicates bias in the studies included, which was not confirmed by Egger’s linear regression test (*P* = .08).

## Methods Used for Diagnosis of *Pseudomonas Aeruginosa*

As listed in Table 2, some studies have used molecular methods, some used phenotypic methods, and some of them used both methods to detect *P. aeruginosa *isolates. Molecular techniques were polymerase chain reaction (PCR),^21,31^ random amplified polymorphic DNA (RAPD)-PCR,^32^ pulsed-field gel electrophoresis (PFGE),^33^ repetitive intergenic consensus (ERIC)-PCR,^34^ allele specific PCR (ASPCR),^3^ sequencing.^5^ Also, phenotypic methods included conventional microbiological tests (oxidase tests, arginine dehydrolase, citrate, OF glucose, catalase test, and sugar fermentation test); culture (blood agar, tryptic soy agar, brain–heart infusion, MacConkey agar, cetrimide agar, Columbia blood agar, Columbia chocolate agar, and chocolate agar); staining (ethidium bromide, Congo red staining, and Gram staining).

### Antibiotic Resistance Patterns

As clear in Table 3, the antibiotic resistance pattern varied among different studies conducted in the Middle East. The fascinating point is that the study conducted by M. S. Ahmed et al from Qatar reported a high prevalence against all antibiotics tested.^30^ M. S. Ahmed et al^30^ reported high resistance of 96.6%, 91.2%, 90.7%, 90.2%, 73.2%, 58%, and 54.6% to cefepime, ciprofloxacin, piperacillin–tazobactam, meropenem, gentamycin, amikacin, and tobramycin, respectively. Studies conducted by Bozkurt-Güzel et al^35^ from Türkiye, and Kodori et al^36^ from Iran, both reported low resistance between 0%-11% against all antibiotics tested. From studies included in Table 3, we can find that there is a slightly high antibiotic resistance in Arabic countries (Jordan, Iraq, and Qatar). The lowest level of resistance of *P. aeruginosa* was toward colistin (0%-13.3%).

As shown in Figure 2, the resistance to tobramycin and aztreonam has increased over time. From 2000 to 2017, the resistance was almost the same, but since 2017, the trend of resistance has changed and the level of resistance has increased gradually. Resistance rate from 3.5%^37^ and 14.3% changed to 87%^38^ and 54.6%^30^ for azteronam and tobramycin in 2020, respectively. Resistance to β-lactams such as Ceftazidime and Piperacillin is available in Table 3, as half of the studies reported high resistance to Ceftazidime, while all studies except one^39^ reported low resistance to Piperacillin. Regarding Amikacin, most studies, resistance was low to moderate resistance, but some studies included here reported a high rate.^30,39,40^

## Discussion

*Pseudomonas aeruginosa *exacerbates the pulmonary disease that frequently occurs in CF patients. Based on the patient registry annual data report in 2019, the prevalence of *P. aeruginosa *continues to decline over time (57% in 2004 to 43% in 2019); this might connect partly to the extensive application of therapy to eliminate early acquisition.^17,41^ As well, in Europe, the overall prevalence of *P. aeruginosa* has meaningfully decreased.^42^ On the contrary, according to our systematic review, the trend of prevalence of* P. aeruginosa* in CF patients. in Middle East countries from 2000 to the end of 2023 did not show any special change. In European countries, the prevalence of chronic Pseudomonas infection is between 14.29% to 62.16%, their results are compared to our findings, where we had a varied prevalence of about 5.9%- 76.2%, and also the combined prevalence of 34.3% for* P. aeruginosa* in Middle East countries.

The rate of infection caused by MDR-PA strains in adults with CF is high, which is probably due to the high exposure of these people to antibiotics. These findings are approximately in accordance with 2 studies included in the present review (about 8%-9%). According to the data extracted in our review, a higher percentage of CF patients were male. In consistency with our data, the patient registry annual data report in 2019 reported the percentage of male patients with CF as follows: 52% in 2004, 51.8% in 2009, 51.6% in 2014, 51.8% in 2018, and 51.8% in 2019. Since in most cases, *P. aeruginosa *is isolated from chronic infection, most studies included in the current review showed that most patients had chronic colonization conditions with *P. aeruginosa*. A full analysis of chronically infected CF patients with* P. aeruginosa* is required because early colonization with this microorganism is connected with higher morbidity and mortality.^5^

Antibiotic resistance is one of the most important concerns of health systems because its presence makes the treatment of diseases more difficult, which increases the mortality rate, the medical costs, and the recovery time in hospitals (WHO, 2018). It also imposes more medical costs on both patients and health systems.^43^

As mentioned in the results, there is a slightly high antibiotic resistance in Arabic countries (Jordan, Iraq, and Qatar). This is referred to the fact that these studies included older children and adults who are exposed to more antibiotics, resulting in increased resistance. Additionally, studies conducted by H. Y. Al Dawodeyh et al,^40^ M. S. Ahmed et al,^30^ and A. Abdul Wahab et al^26^ reported some MDR strains, which naturally show more resistance to antibiotics.^44^ As a result, we can conclude that there is no significant difference in antibiotic resistance among the countries included in the present review from the Middle East.

Recently, there are 3 classes of inhaled antibiotics including tobramycin, aztreonam, and colistin that are used for the treatment of infection resulting from* P. aeruginosa *in CF patients. Among them, tobramycin is used most commonly, followed by aztreonam and colistin.^45^ This should be noted, their peak use for all 3 antibiotics is between youth and adulthood.

As well, Dornase alfa and hypertonic saline both are widely used for patients with CF. In addition, people with *P. aeruginosa* are prescribed Azithromycin with its peak use at slightly older ages.^44^ As shown in our results, the resistance to tobramycin and aztreonam antibiotics has increased over time.^30,38,39^ The lowest antibiotic resistance was reported against colistin. We think the low level of resistance to colistin in the present review is perhaps due to the fact that in 2013, the CF Foundation Pulmonary Guidelines Committee stated that there is not enough evidence for the role of colistin and some other medications in decreasing the severity of lung disease, improving lung function, or improving quality of life in people with cystic fibrosis, so the use of it in CF patients in the Middle East and other parts of the world has recently decreased.^46^ However, tobramycin, aztreonam, and colistin are the therapeutic options recommended by the European Cystic Fibrosis Society for the treatment of chronic *P. aeruginosa *infection.^47^

The antibiotic resistance pattern varies against different antibiotics in various studies conducted in the Middle East. It should be taken into consideration that the study conducted by M. S. Ahmed et al^30^ from Qatar reported a high prevalence against all antibiotics tested. M. S. Ahmed et al^30^ reported high resistance of 96.6%, 91.2%, 90.7%, 90.2%, 73.2%, 58%, and 54.6% to cefepime, ciprofloxacin, piperacillin–tazobactam, meropenem, gentamycin, amikacin, and tobramycin, respectively,^3^ in contrast, studies conducted by Bozkurt-Güzel et al^35 ^from Türkiye S. Mamishi et al^48^ and Kodori et al^36^ both from Iran, reported low resistance between 0% and 11% against all antibiotics tested. This is likely is because of the source of bacterial isolates, year of study, location, and infection control measures in each country or region.^39^ Also, an important note is that CF patients from years ago weren’t the same as those in 2022, owing to advances in pharmacotherapy and the care they have taken over time.^49^

Cystic Fibrosis Foundation, alongside tobramycin, aztronam, and colistin, makes a list of commonly prescribed intravenous antibiotics to encounter infections caused by *P. aeruginosa *in CF patients. Those are penicillin (piperacillin), cephalosporins (ceftazidime, cefepime, ceftolozane), carbapenems (meropenem, imipenem, doripenem), aminoglycosides (amikacin, gentamycin), a macrolide (azithromycin), and fluoroquinolones (ciprofloxacin, levofloxacin) (https://www.cff.org/Life-With-CF/Treatments-and Therapies/Medications/Antibiotics/).

Ceftazidime is commonly the best cephalosporin used to reduce acute pulmonary exacerbations in CF patients, chiefly owing to known bactericidal activity against *P. aeruginosa *compared to other cephalosporins,^50,51^ similarly, piperacillin has the same sufficient activity against *P. aeruginosa* as ceftazidime is.^52^ Amikacin is an alternative antibiotic used in cases where CF patients are unable to tolerate tobramycin or who are infected by *P. aeruginosa* strains resistant to tobramycin.^53^ According to our findings, half of the studies reported high resistance to ceftazidime (β-lactam) compared to all studies except one^39^ reported low resistance to piperacillin (β-lactam). Regarding amikacin, most studies, resistance was low to moderate resistance, but some studies included here reported a high rate.^30,39,40^

Our study showed that the pattern of antibiotic resistance is almost the same in the countries included from the Middle East. In some studies, the resistance pattern was higher; it depends on the site of study, year of study, infection control policies, socioeconomic status, and type of isolates. Resistance against inhaled first-choice antibiotics, including tobramycin and aztreonam, and intravenous antibiotic (amikacin), has increased gradually over time. Of course, colistin, piperacillin, and ticarcillin are the most effective antibiotics for treating* P. aeruginosa *infection in CF patients according to our findings.

It can be concluded from our study that the prevalence of* P. aeruginosa *and antibiotic resistance is high. However, it should be noted that some studies are small and there were no studies from some countries in the region included in this review, so in light of these points the interpretation of the results must be carefully considered. Our study showed that colistin and ticarcillin were found to be the best antibiotics for decreasing postantibiotic effects (PAEs) in CF patients from the Middle East. Therefore, physicians should pay more attention to therapeutic protocols to prevent further resistance, and antibiotic prescriptions must be based on the results of antibiotic susceptibility testing.

### Limitations

The main limitations of this systematic review and meta-analysis are: the present review only included studies that were published in English. Unpublished studies and studies from other languages such as Arabic, Persian, and Turkish were not included. The small sample size of some studies and the lack of studies from some Middle Eastern countries for inclusion in this review as well; epidemiologic data differ significantly among countries with different socioeconomic statuses, which has resulted in wide differences in the findings that need to be evaluated more carefully.

## Data Availability Statement

The data that support the findings of this study are available on request from the corresponding author.

## Figures and Tables

**Table 1.** Characteristics of Studies Included in the Present Review

Study	Publication Year	Years of Study	Sample Size	Location	Type of Study	*P. aeruginosa* Frequency n (%)	Source of Samples
B. Şener^32^	2001	1991-2000	248	Türkiye	Cross-sectional	130 (52.4 )	Sputa or deep throat swab
K. Kakish^5^	2001	1995-2000	72	Jordan	Prospective cohort	12 (30)	Sputum cultures
F. Eftekhar^31^	2003	–	64	Iran	Cross-sectional	21 (32.8)	Sputum samples or throat swabs
H. Nazik^8^	2007	2003	60	Türkiye	Retrospective cohort	49 (81.66)	Sputum
F. Eftekhar^33^	2009	2004-2005	46	Iran	Cross-sectional	31 (67.4)	Sputum
Z. Movahedi^34^	2013	2010	49	Iran	Cross-sectional	16 (32.65)	Different sources
M. M. El-Falaki^3^	2013	2010-2012	36	Egypt	Longitudinal	8 (22)	sputum
D. Dogru^54^	2013	2008-2010	90	Türkiye	Cross-sectional	56 (62.2)	Sputum, oropharyngeal swabs or BAL
M. Douraghi^21^	2014	2012-2011	100	Iran	Cross-sectional	40 (40)	Sputum samples or throat swabs
M. S. Ahmed^30^	2019	2014-2015	205	Qatar	Cross-sectional study	12 (6.9)	Different sources
A. S. Sasihuseyinoglu^55^	2019	2011- 2016	28	Türkiye	Retrospective cohort study	7 (25)	sputum
A. M. Ali^38^	2020	2018	17	Iraq	Cross-sectional	8 (47)	different sources
D. Dogru^56^	2020	Until 2017	1170	Türkiye	Cross‐sectional	245 (20.94)	-
U. Aslanhan^57^	2021	–	16	Türkiye	–	8(50)	Sputum
M. Kodori^36^	2021	2015- 2017	153	Iran	Descriptive cross‐sectional	83 (55.5)	Sputum
E. Fidan^58^	2021	2015-2018	1767	Türkiye	–	1034 (58.4)	Respiratory samples
H. H. Mursaloglu^59^	2021	2015-2019	309	Türkiye	Retrospective cross-sectional	120/309 (38.8)	Sputum and pharyngeal swab
A. AbdulWahab^60^	2021	2020	21	Qatar	Prospective cross-sectional	16/21	Sputum
A. G. Abdul-Qadir^61^	2021	–	31	Iraq	Cross-sectional	6/31 (19.3%)	Sputum
R. Al-Baba^62^	2021	2007-2017	173	Syria	Retrospective cross-sectional	12/173 (6.9)	Bronchial
T. R.Gursoy^63^	2022	2018	359	Türkiye	Cross-sectional	33/359 (9.2)	–
H. Banjar^64^	2022	1990-2018	305	Saudi Arabia	–	120 (34.9)	Respiratory samples
S. Mamishi^48^	2022	–	534	Iran	Cross-sectional	(18%) 94	Sputum
N. Alshraiedeha^65^	2022	2018-2019	43	Jordan	–	17 (39.53)	Pharyngeal swabs
S. Erfanimanesh^66^	2022	2018-2019	129	Iran	Cross-sectional	43 (41.7)	Sputum + throat swab BAL
A. İ. Yılmaz^67^	2023	2011-2021	87	Türkiye	Retrospective cross-sectional	32/87 (36.8)	–
S. M. Goudarzi^68^	2023	2018-2019	121	Iran	–	32/121 (26.5)	Pharyngeal swabs
F. Khani Nozari^69^	2023	2015-2018	76	Iran	Cross-sectional	22/76 (29)	Sputum
**Study**		**Age**		**MDR Isolates (%)**	**Genus**		**Patient Condition**
					**Female (%)**	**Male (%)**	
B. Şener		3-21 years (mean: 11.17)^1^		–	35	65	Chronic
K. S. Kakish		3-96 months (mean: 30.7) pediatrics		–	48.62	51.38	Chronic
F. Eftekhar 2003		2 months-18 years children		9.5	58	42^2^	Chronic
H. Nazik		–		–	–	–	Chronic
F. Eftekhar 2009		3 months-23 years		–	41.30	58.69	Chronic
Z. Movahedi		5-192 month Children		–	–	–	–
M. M. El-Falaki		2 month-16 years Children		–	39	61	Chronic
D. Dogru 2013		8 month-26.3 years		–	50	50	Chronic, intermittent, negative, and mucoid groups
M. Douraghi		1-23 years		–	54	46	–
M. S. Ahmed		<14-65 years		8.1	25.4	74.6	–
A. S. Sasihuseyinoglu		Mean: 6.8 years Children		–	32	68	–
A. M. Ali		– –		–	–	–	–
D. Dogru (2020)		3-42 years		–	46	54	Chronic
U. Aslanhan		0-16 years		–	50	50	–
Kodori, M		Mean: 2.93 years Children		–	48	52	–
S. Erfanimanesh		Mean: 9.41 ± 6.33 Children		25.5	45 (53.4)	40 (46.6)	Chronic
A. AbdulWahab		24.29 ± 5.28		–	8	13	Chronic
A. İ. Yılmaz		4.2 (1-12)		–	41 (47.1)	46 (52.9)	Chronic
S. M. Goudarzi		–		–	–	–	Chronic
F. Khani Nozari		14-16		–	31 (40.7)	45 (59.2)	Chronic
T.R. Gursoy		–			179(49.9)	180 (50.1)	Chronic
A. G. Abdul-Qadir		4.6 ± 4.02		–	13 (41.94%)	18 (58.06%)	Chronic
R. Al-Baba		Children 3.4 ± 8.5		–	92/173	81/173	Chronic

BAL, bronchoalveolar lavage; MDR, multidrug-resistant; *P. aeruginosa, Pseudomonas aeruginosa.*

^1^From table.

^2^Estimated from figure.

**Table 2.** Diagnostic Methods Used in the Diagnosis of *Pseudomonas aeruginosa* Isolates

Study	Publication Year	Diagnostic Method	Phenotypic Tests		
			Oxidase Tests OF Glucose	-	Ethidium Bromide
B. Şener^32^	2001	RAPD-PCR	-	-	-
K. S. Kakish^5^	2001	Sequencing		Blood agar	Ethidium bromide
F. Eftekhar^31^	2003	PCR	-	Tryptic soy agar	Ethidium bromide
H. Nazik^8^	2007	RAPD-PCR	-	Tryptic soy agar	Ethidium bromide
F. Eftekhar^33^	2009	RAPD-PCR PFGE	Oxidase production	BHI	Ethidium bromide
Z. Movahedi^34^	2013	ERIC-PCR	-	-	-
M. M. El-Falaki^3^	2013	PCR (ASPCR)	-	Sputum culture	-
D. Dogru^54^	2013	-		Sputum culture	-
M. Douraghi^21^	2014	PCR	Oxidase, citrate, OF glucose, and arginine dihydrolase	Cetrimide agar, blood agar, and MacConkey agar	Ethidium bromide
M. S. Ahmed^30^	2019	-	-	-	-
A. S. Sasihuseyinoglu^55^	2019	-	Pulmonary function tests Radiological study	Sputum cultures	-
A. M. Ali^38^	2020	Gradient PCR ERIC-PCR	Oxidase test catalase test microtiter plate. Biofilm Formation Assay	BHI	Congo red stain Gram stain
D. Dogru^56^	2020	Sweat tests	-	-	-
U. Aslanhan^57^	2021	-	Oxidative burst, phagocytic, chemotactic index	Short‐term whole blood cultures	Annexin V/propidium iodide Antibodies and intracytoplasmic cytokine staining
M. Kodori^36^	2021	Sweat tests	Biofilm Formation Assay	Blood agar chocolate agar MacConkey agar	Crystal violet
E. Fidan^58^	2021	Laboratory operating systems retrospectively	-	Sputum cultures	-
H.Banjar^64^	2021	Laboratory operating systems retrospectively	-	Sputum cultures	-
S. Mamishi^48^	2022	Random amplified polymorphic DNA polymerase chain reaction	-	-	-
N. Alshraiedeha^65^	2022	Laboratory operating systems retrospectively	-	Sputum cultures	-
S. Erfanimanesh	2022	PCR	-	Sputum cultures	-
A. AbdulWahab	2021	-	-	Sputum cultures	-
S. M. Goudarzi	2023	Nested-PCR sequencing	Standard biochemical tests	Blood agar, MacConkey agar, colonial morphology, Gram staining	Standard biochemical tests

ASPCR, allele specific polymerase chain reaction; BHI, brain–heart infusion; CDDT, combined double disk synergy test; CRA, Congo red agar; ERIC-PCR, repetitive intergenic consensus-polymerase chain reaction; MHA, Mueller–Hinton agar; MHT, modified Hodge test; MIC, minimum inhibitory concentration; PCR, polymerase chain reaction; PFGE, pulsed-field gel electrophoresis; RAPD, random amplified polymorphic DNA; TCP, tissue culture plate; TSI, triple sugar iron agar.

**Table 3.** Antibiotics Resistance Patterns in *Pseudomonas Aeruginosa* Isolated from Cystic Fibrosis Patients

Study	Antibiotics (n) %																	
	AMK	GEN	AZT	IMP	TOB	PIP	TIC	CIP	CEF	CAR	CFT	MER	CFP	PIP+TAZ	COL	TER+SUL	CFO	LEV
Eftekhar(2003)^31^	19 (4)	38 (8)	-	0 (0)	14.3 (3)	19 (4)	24 (5)	9.5 (2)	14.3 (3)	57 (12)	-	-	-	19 (4)	0 (0)	-	-	-
Ghazi (2012)^37^	3.5 (2)	18 (8)	3.5 (2)	10 (4)	3.5 (2)	0(0)	3.9 (2)	21 (9)	-	-	0	7 (3)	0	0	-	96.5 (42)	-	-
Bozkurt-Güzel (2012)^35^	0 (0)	-	-	-	-	-	-	-	-	-	10 (1)	0 (0)	-	-	0 (0)	-	-	-
Movahedi (2013)^34^	37.5 (6)	43 (7)	-	43 (7)	-	-	-	43 (7)	-	56.2 (9)	75 (12)	-	87.5 (14)	37.5 (6)	-	-	-	-
Tabatabaee (2013)^2^	2.2 (1)	6.7 (2)	-	73.3 (22)	0 (0)	2.2 (1)	8.9 (3)	0 (0)	-	20 (6)	88.9 (28)	-	-	-	13.3 (4)	-	51.1 (16)	-
Rafiee (2016)^70^	4 (2)	-	4 (2)	10 (5)	4 (2)	2 (1)	6 (3)	-	22 (11)	8 (4)	2 (1)	2 (1)	-	-	-	-	-	-
Tarhani (2016)^71^	-	43.3 (13)	26.6 (8)	3.3 (1)	-	10 (3)	-	16.6 (5)	-	-	33.3 (10)	10	16.6	-	-	-	-	-
Abdul Wahab (2017)^26^	32.8 (20)	41 (25)	-	-	-	-	-	23 (14)	-	-	-	11.5 (7)	29.5 (18)	9.8 (6)	-	-	-	-
A.Pournaja (2018)^72^	16.8 (24)	23.1 (33)	4.9 (7)	6.3 (9)	-	5.6 (8)	0	28.7 (41)	-	-	23.1 (33)	-	11.9 (17)	0 (0)	0 (0)	-	-	10.5 (15)
H. Y. Al Dawodeyh (2018)^40^	50.9 (31)	62.3 (38)	42.7 (26)	19.7 (12)	-	-	-	50.9 (31)	18 (11)	-	-	21.3 (13)	18 (11)	37.8 (23)	0 (0)	-	75.4 (46)	-
Talebi (2019)^39^	53.65 (22)	39.02 (16)	43.9 (18)	46.34 (19)	-	41.46 (17)	-	46.34 (19)	-	-	46.34 (19)	-	51.21 (21)	34.14 (14)	-	-	-	-
M. S. Ahmed (2019)^30^	58 (7)	73.2 (9)	-	-	54.6 (6)	-	-	91.2 (11)	-	-	-	90.2 (10)	96.6 (11)	90.7 (10)	3.4 (1)	-	-	-
A. M. Ali (2020)^38^	-	-	87 (7)	10 (1)	32 (3)	-	-	55 (4)	67 (5)	-	78 (6)	-	77 (6)	71 (6)	-	71 (6)	-	22 (2)
Kodori (2021)^36^	7.05 (6)	11.7 (10)	-	7.05 (6)	-	-	-	-	-	-	5.9 (5)	-	-	2.3 (2)	0 (0)	-	-	-
S.Mamishi (2022)^48^	-	-	-	5 (5%)	-	3 (3.5%)	-	-	-	-	3 (3.5%)	-	-	-	-	-	-	-
N. Alshraiedeha(2022)^65^	1 (7%)	2 (11.8%)	-	4 (21%)	4 (21%)	-	-	1	-	-	0	-	-	-	-	-	-	0
S. M. Goudarzi	-	25.8	-	7.1	0	-	-	3.3	-	-	-	-	-	-	-	-	-	-

AMK, amikacin; AZT, aztreonam; CAR, carbenicillin; CEF, ceftriaxone; CFO, cefotaxime; CFP, cefepime; CFT, ceftazidime; CIP, ciprofloxacin; CLO, cloxacillin; COL, colistin; GEN, gentamycin;IMP, Imipenem; LEV, levofloxacin; PIP, piperacillin; MER, meropenem; TER+SUL, trimethoprim + sulfamethoxazole; TOB, tobramycin; TIC, ticarcillin.

## References

[1] Alavi Foumani A Yaghubi Kalurazi T Mohammadzadeh Rostami F Sedigh Ebrahim-Saraie H Nazari-Alam A Halaji M . Epidemiology of Pseudomonas aeruginosa in cystic fibrosis patients in Iran: A systematic review and meta-analysis. Infez Med. 2020;28(3):314 321. 32920566

[2] Tabatabaee SA Nariman S Taghipour R , et al. Antibiogram and genotype of Pseudomonas aeroginosa in cystic fibrosis. Stud Med Sci. 2013;24(3):184 192.

[3] El-Falaki MM Shahin WA El-Basha NR Ali AA Mehaney DA El-Attar MM . Profile of cystic fibrosis in a single referral center in Egypt. J Adv Res. 2014;5(5):563 568. (10.1016/j.jare.2013.07.005) 25685524 PMC4294314

[4] Iwuji K Larumbe-Zabala E Bijlani S , et al. Prevalence of bactericidal/permeability-increasing protein autoantibodies in cystic fibrosis patients: systematic review and meta-analysis. Pediatr Allergy Immunol Pulmonol. 2019;32(2):45 51. (10.1089/ped.2018.0970) 31508255 PMC6733050

[5] Kakish KS . Cystic fibrosis in Jordan: clinical and genetic aspects. Bahrain Med Bull. 2001;23(4):157 159.

[6] Hayes D Tobias JD Mansour HM , et al. Pulmonary hypertension in cystic fibrosis with advanced lung disease. Am J Respir Crit Care Med. 2014;190(8):898 905. (10.1164/rccm.201407-1382OC) 25222938

[7] Goetz D Ren CL . Review of cystic fibrosis. Pediatr Ann. 2019;48(4):e154 e161. (10.3928/19382359-20190327-01) 30986316

[8] Nazik H Ongen B Erturan Z Salcioğlu M . Genotype and antibiotic susceptibility patterns of Pseudomonas aeruginosa and Stenotrophomonas maltophilia isolated from cystic fibrosis patients. Jpn J Infect Dis. 2007;60(2-3):82 86. (10.7883/yoken.JJID.2007.82) 17515637

[9] Máiz L Girón RM Olveira C , et al. Inhaled antibiotics for the treatment of chronic bronchopulmonary Pseudomonas aeruginosa infection in cystic fibrosis: systematic review of randomised controlled trials. Expert Opin Pharmacother. 2013;14(9):1135 1149. (10.1517/14656566.2013.790366) 23586963

[10] Abdul Wahab AA Taj-Aldeen SJ Hagen F , et al. Genotypic diversity of Pseudomonas aeruginosa in cystic fibrosis siblings in Qatar using AFLP fingerprinting. Eur J Clin Microbiol Infect Dis. 2014;33(2):265 271. (10.1007/s10096-013-1954-1) 23996049

[11] Kidd TJ Ramsay KA Vidmar S , et al. Pseudomonas aeruginosa genotypes acquired by children with cystic fibrosis by age 5-years. J Cyst Fibros. 2015;14(3):361 369. (10.1016/j.jcf.2014.12.007) 25563522

[12] Karami P Khaledi A Mashoof RY , et al. The correlation between biofilm formation capability and antibiotic resistance pattern in Pseudomonas aeruginosa. Gene Rep. 2020;18:100561. (10.1016/j.genrep.2019.100561)

[13] Ghazalibina M Morshedi K Farahani RK Babadi M Khaledi A . Study of virulence genes and related with biofilm formation in Pseudomonas aeruginosa isolated from clinical samples of Iranian patients: a systematic review. Gene Rep. 2019;17:100471. (10.1016/j.genrep.2019.100471)

[14] Heidarzadeh S Enayati Kaliji Y Pourpaknia R , et al. A meta-analysis of the prevalence of class 1 integron and correlation with antibiotic resistance in Pseudomonas aeruginosa recovered from Iranian burn patients. J Burn Care Res. 2019;40(6):972 978. (10.1093/jbcr/irz135) 31326983

[15] Esmaeili D Daymad SF Neshani A Rashki S Marzhoseyni Z Khaledi A . Alerting prevalence of MBLs producing Pseudomonas aeruginosa isolates. Gene Rep. 2019;16:100460. (10.1016/j.genrep.2019.100460)

[16] Hadadi-Fishani M Khaledi A Fatemi-Nasab ZS . Correlation between biofilm formation and antibiotic resistance in Pseudomonas aeruginosa: a meta-analysis. Infez Med. 2020;28(1):47 54. 32172260

[17] Mogayzel PJ Naureckas ET Robinson KA , et al. Cystic Fibrosis Foundation pulmonary guideline. Pharmacologic approaches to prevention and eradication of initial Pseudomonas aeruginosa infection. Ann Am Thorac Soc. 2014;11(10):1640 1650. (10.1513/AnnalsATS.201404-166OC) 25549030

[18] Wainwright CE Elborn JS Ramsey BW , et al. Lumacaftor–ivacaftor in patients with cystic fibrosis homozygous for Phe508del CFTR. N Engl J Med. 2015;373(3):220 231. (10.1056/NEJMoa1409547) 25981758 PMC4764353

[19] Schwarz C Schulte-Hubbert B Bend J , et al. S3-Leitlinie: Lungenerkrankung bei Mukoviszidose–Modul 2: Diagnostik und Therapie bei der chronischen Infektion mit Pseudomonas aeruginosa. Pneumologie. 2018;72(5):347 392. (10.1055/s-0044-100191) 29758578

[20] Karballaei Mirzahosseini H Hadadi-Fishani M Morshedi K Khaledi A . Meta-analysis of biofilm formation, antibiotic resistance pattern, and biofilm-related genes in Pseudomonas aeruginosa isolated from clinical samples. Microb Drug Resist. 2020;26(7):815 824. (10.1089/mdr.2019.0274) 31976811

[21] Douraghi M Ghasemi F Dallal MM Rahbar M Rahimiforoushani A . Molecular identification of Pseudomonas aeruginosa recovered from cystic fibrosis patients. J Prev Med Hyg. 2014;55(2):50 53. 25916020 PMC4718328

[22] Ramirez MS Tolmasky ME . Aminoglycoside modifying enzymes. Drug Resist Updat. 2010;13(6):151 171. (10.1016/j.drup.2010.08.003) 20833577 PMC2992599

[23] Hosseini SMJ Naeini NS Khaledi A Daymad SF Esmaeili D . Evaluate the relationship between class 1 integrons and drug resistance genes in clinical isolates of Pseudomonas aeruginosa. Open Microbiol J. 2016;10:188 196. (10.2174/1874285801610010188) 28077975 PMC5204062

[24] Karimi E Ghalibafan F Esfandani A , et al. Antibiotic resistance pattern in Pseudomonas aeruginosa isolated from clinical samples other than burn samples in Iran. Avicenna J Med Biotechnol. 2021;13(1):35 41. (10.18502/ajmb.v13i1.4575) 33680371 PMC7903437

[25] Aghazadeh M Rezaee MA Nahaei MR , et al. Dissemination of aminoglycoside-modifying enzymes and 16S rRNA methylases among Acinetobacter baumannii and Pseudomonas aeruginosa isolates. Microb Drug Resist. 2013;19(4):282 288. (10.1089/mdr.2012.0223) 23577624

[26] AbdulWahab A Zahraldin K Sid Ahmed MAS , et al. The emergence of multidrug-resistant Pseudomonas aeruginosa in cystic fibrosis patients on inhaled antibiotics. Lung India Off Organ Indian Chest Soc. 2017;34(6):527 531. (10.4103/lungindia.lungindia_39_17) PMC5684810 29098998

[27] Döring G Conway SP Heijerman HG , et al. Antibiotic therapy against Pseudomonas aeruginosa in cystic fibrosis: a European consensus. Eur Respir J. 2000;16(4):749 767. (10.1034/j.1399-3003.2000.16d30.x) 11106223

[28] Flume PA O'Sullivan BP Robinson KA , et al. Cystic fibrosis pulmonary guidelines: chronic medications for maintenance of lung health. Am J Respir Crit Care Med. 2007;176(10):957 969. (10.1164/rccm.200705-664OC) 17761616

[29] Munn Z Moola S Lisy K Riitano D Tufanaru C . Methodological guidance for systematic reviews of observational epidemiological studies reporting prevalence and cumulative incidence data. Int J Evid-Based Healthc. 2015;13(3):147 153. (10.1097/XEB.0000000000000054) 26317388

[30] Ahmed MS Hassan A Jarir SA , et al. Emergence of multidrug-and pandrug-resistant Pseudomonas aeruginosa from five hospitals in Qatar. Infect Prev Pract. 2019;1(3-4):100027. 34368684 10.1016/j.infpip.2019.100027 PMC8336314

[31] Eftekhar F Khodadad A Henry D Speert D . Isola tion and Genetic Fingerprinting of Pseudomonas aeruginosa from Iranian Patients with Cystic Fibrosis Using RAPD-PCR; 2003:1 (2):95 100

[32] Şener B Köseoğlu O Özçelik U Kocagöz T Günalp A . Epidemiology of chronic Pseudomonas aeruginosa infections in cystic fibrosis. Int J Med Microbiol. 2001;291(5):387 393. (10.1078/1438-4221-00144) 11727823

[33] Eftekhar F Hosseinkhan N Asgharzadeh A Tabatabaei A . Gene tic Profiling of Pseudomonas aeruginosa Isolates from Iranian Patients with Cystic Fibrosis Using RAPD-PCR and PFGE; 2009;12 (3-4):126 132.

[34] Movahedi Z Pourakbari B Mahmoudi S , et al. Pseudomonas aeruginosa infection among cystic fibrosis and ICU patients in the referral children medical hospital in Tehran, Iran. J Prev Med Hyg. 2013;54(1):24 28. 24397002 PMC4718363

[35] Bozkurt-Güzel C Gerçeker AA . Post-antibiotic effect of colistin, alone and in combination with amikacin, on Pseudomonas aeruginosa strains isolated from cystic fibrosis patients. J Antibiot (Tokyo). 2012;65(2):83 86. (10.1038/ja.2011.101) 22126897

[36] Kodori M Nikmanesh B Hakimi H Ghalavand Z . Antibiotic susceptibility and biofilm formation of bacterial isolates derived from pediatric patients with cystic fibrosis from Tehran, Iran. Arch Razi Inst. 2021;76(2):397 406. (10.22092/ari.2020.128554.1416) 34223738 PMC8410193

[37] Ghazi M Khanbabaee G Fallah F , et al. Emergence of Pseudomonas aeruginosa cross-infection in children with cystic fibrosis attending an Iranian referral pediatric center. Iran J Microbiol. 2012;4(3):124 129. 23066486 PMC3465537

[38] Ali AM Al-Kenanei KA Hussein SN Bdaiwi QO . Molecular study of some virulence genes of Pseudomonas aeruginosa isolated from different infections in hospitals of Baghdad. Rev Med Microbiol. 2020;31(1):26 41. (10.1097/MRM.0000000000000194)

[39] Talebi G Hakemi-Vala M . Survey on some carbapenems and colistin resistance genes among Pseudomonas aeruginosa isolates from burn and cystic fibrosis patients, Tehran, Iran. Arch Clin Infect Dis. 2019;14(5). (10.5812/archcid.93651)

[40] Al Dawodeyah HY Obeidat N Abu-Qatouseh LF Shehabi AA . Antimicrobial resistance and putative virulence genes of Pseudomonas aeruginosa isolates from patients with respiratory tract infection. GERMS. 2018;8(1):31 40. (10.18683/germs.2018.1130) 29564246 PMC5845973

[41] Saiman L Siegel JD LiPuma JJ , et al. Infection prevention and control guideline for cystic fibrosis: 2013 update. Infect Control Hosp Epidemiol. 2014;35(suppl 1):S1 S67. (10.1086/676882) 25025126

[42] Hatziagorou E Orenti A Drevinek P , et al. Changing epidemiology of the respiratory bacteriology of patients with cystic fibrosis–data from the European cystic fibrosis society patient registry. J Cyst Fibros. 2020;19(3):376 383. (10.1016/j.jcf.2019.08.006) 31492646

[43] Sydnor ER Perl TM . Hospital epidemiology and infection control in acute-care settings. Clin Microbiol Rev. 2011;24(1):141 173. (10.1128/CMR.00027-10) 21233510 PMC3021207

[44] Marshall B Faro A Fink A . Cystic Fibrosis Foundation Patient Registry. Annual Data Report. Bethesda, MD: Cystic Fibrosis Foundation 2017. Available at: https://www.cff.org/Research/Researcher-Resources/Patient-Registry/2017-Patient-Registry-Annual-Data-Report pdf.

[45] Bodnár R Mészáros Á Oláh M Ágh T . Inhaled antibiotics for the treatment of chronic Pseudomonas aeruginosa infection in cystic fibrosis patients: challenges to treatment adherenceand strategies to improve outcomes. Patient Preference Adherence. 2016;10:183 193. (10.2147/PPA.S53653) 26937178 PMC4762437

[46] Mogayzel PJ Naureckas ET Robinson KA , et al. Cystic fibrosis pulmonary guidelines: chronic medications for maintenance of lung health. Am J Respir Crit Care Med. 2013;187(7):680 689. (10.1164/rccm.201207-1160oe) 23540878

[47] Castellani C Duff AJA Bell SC , et al. ECFS best practice guidelines: the 2018 revision. J Cyst Fibros. 2018 revision. 2018;17(2):153 178. (10.1016/j.jcf.2018.02.006) 29506920

[48] Mamishi S Akhlaghi A Pourakbari B , et al. Ant imicrobial susceptibility and genotyping of microorganisms isolated from sputum culture of children with cystic fibrosis in an Iranian referral Children’s Hospital. Wien Med Wochenschr. 2023;173 (3):1 6. 10.1007/s10354-022-00970-x 36167900

[49] El Hassani M Caissy JA Marsot A . Antibiotics in adult cystic fibrosis patients: a review of population pharmacokinetic analyses. Clin Pharmacokinet. 2021;60(4):447 470. (10.1007/s40262-020-00970-3) 33447944

[50] Yong J Frost F Nazareth D Walshaw M . Case report: haemolytic anaemia with ceftazidime use in a patient with cystic fibrosis. F1000Res. 2018;7:475. (10.12688/f1000research.14505.1) 29770214 PMC5920311

[51] Petri W . Penicillins, Cephalosporins, and Other β-Lactam Antibiotics. Goodman and Gilman's the Pharmacological Basis of Therapeutics. 12th ed. New York: McGraw-Hill; 2011:1477 1504.

[52] Bulitta JB Duffull SB Kinzig-Schippers M , et al. Systematic comparison of the population pharmacokinetics and pharmacodynamics of piperacillin in cystic fibrosis patients and healthy volunteers. Antimicrob Agents Chemother. 2007;51(7):2497 2507. (10.1128/AAC.01477-06) 17485505 PMC1913222

[53] Illamola SM Huynh HQ Liu X , et al. Population pharmacokinetics of amikacin in adult patients with cystic fibrosis. Antimicrob Agents Chemother. 2018;62(10):e00877-18. (10.1128/AAC.00877-18) 30061295 PMC6153835

[54] Doğru D Pekcan S Yalçın E , et al. The role of serum Pseudomonas aeruginosa antibodies in the diagnosis and follow-up of cystic fibrosis. TJP. Turk J Pediatr. 2013;55(1):50 57. 23692832

[55] Sasihuseyinoglu AS Altıntaş DU Soyupak S , et al. Evaluation of high resolution computed tomography findings of cystic fibrosis. Korean J Intern Med. 2019;34(2):335 343. (10.3904/kjim.2017.287) 29976036 PMC6406085

[56] Dogru D Çakır E Şişmanlar T , et al. Cystic fibrosis in Turkey: first data from the national registry. Pediatr Pulmonol. 2020;55(2):541 548. (10.1002/ppul.24561) 31710166

[57] Aslanhan U Cakir E Pur Ozyigit L , et al. Pseudomonas aeruginosa colonization in cystic fibrosis: Impact on neutrophil functions and cytokine secretion capacity. Pediatr Pulmonol. 2021;56(6):1504 1513. (10.1002/ppul.25294) 33512090

[58] Fidan E Alci G Koldaş SS , et al. Cumulative antimicrobial susceptibility data of Pseudomonas aeruginosa isolates from cystic fibrosis patients: 4-year experience. J Pediatr Infect Dis. 2021;16(5):242 246. (10.1055/s-0041-1731344)

[59] Mursaloglu HH Akın C Yılmaz Yeğit C , et al. Comparison of intravenous and non‐intravenous antibiotic regimens in eradication of P. aeruginosa and MRSA in cystic fibrosis. Pediatr Pulmonol. 2021;56(12):3745 3751. (10.1002/ppul.25646) 34436829

[60] AbdulWahab A Allangawi M Thomas M , et al. Sputum and plasma neutrophil elastase in stable adult patients with cystic fibrosis in relation to chronic Pseudomonas aeruginosa colonization. Cureus. 2021;13(6):e15948. (10.7759/cureus.15948) 34221778 PMC8238017

[61] Abdul-Qadir AG Al-Musawi BM Thejeal RF . Al-Omar SA-B. Molecular analysis of CFTR gene mutations among Iraqi cystic fibrosis patients. Egypt J Med Hum Genet. 2021;22:1 7. 38624701 10.1186/s43042-021-00164-x PMC8110311

[62] Al-Baba R Zetoune AB . A retrospective study of cases diagnosed with cystic fibrosis at a single care center in Syria. Egypt J Med Hum Genet. 2021;22(1):1 11.38624675

[63] Ramasli Gursoy T Aslan AT Asfuroglu P , et al. Clinical findings of patients with cystic fibrosis according to newborn screening results. Pediatr Int. 2022;64(1):e14888. (10.1111/ped.14888) 34131975

[64] Banjar H Ghawi A AlMogarri I , et al. First report on the prevalence of bacteria in cystic fibrosis patients (CF) in a tertiary care center in Saudi Arabia. Int J Pediatr Adolesc Med. 2022;9(2):108 112. (10.1016/j.ijpam.2021.07.001) 35663786 PMC9152558

[65] Alshraiedeh N Atawneh F Bani-Salameh R Alsharedeh R Al Tall Y Alsaggar M . Identification and characterization of bacteria isolated from patients with cystic fibrosis in Jordan. Ann Med. 2022;54(1):2796 2804. (10.1080/07853890.2022.2131282) 36264155 PMC9586617

[66] Erfanimanesh S Emaneini M Modaresi MR , et al. Distribution and characteristics of bacteria isolated from cystic fibrosis patients with pulmonary exacerbation. Can J Infect Dis Med Microbiol. 2022;2022:5831139. (10.1155/2022/5831139) 36593975 PMC9805393

[67] Yılmaz Aİ Pekcan S Eyüboğlu TŞ , et al. Negle cted Children with Cystic Fibrosis Due to War (Turkey Profile of Refugee Patients); 2023;23 (2):22 29.

[68] Moazami Goudarzi S Shahpouri Arani Y Abdi Ali A , et al. Comparison of culture and PCR-DGGE methods to evaluate the airways of cystic fibrosis patients and determination of their antibiotic resistance profile. Iran J Microbiol. 2023;15(6):750 758. (10.18502/ijm.v15i6.14135) 38156302 PMC10751606

[69] Khani Nozari FK Modaresi M Allahverdi B Shirzadi R Fattahi M . Association between sputum culture results and pulmonary changes in children with cystic fibrosis. Iran J Microbiol. 2023;15(6):759 764. (10.18502/ijm.v15i6.14136) 38156305 PMC10751615

[70] Rafiee R Eftekhar F Tabatabaei SA Minaee-Tehrani D . Detection of AmpC and extended-spectrum beta-lactamases in clinical isolates of Pseudomonas aeruginosa from patients with cystic fibrosis [Journal]. mljgoums. 2016;10(3):28 32. (10.18869/acadpub.mlj.10.3.28)

[71] Tarhani M Hakemi-Vala M Hashemi A Nowroozi J Khanbababee G . Detection of metallo-β-lactamases and Klebsiella pneumonia carbapenemases in Pseudomonas aeruginosa isolates from cystic fibrosis patients. Arch Pediatr Infect Dis. 2016;4(3). (10.5812/pedinfect.35905)

[72] Pournajaf A Razavi S Irajian G , et al. Integron types, antimicrobial resistance genes, virulence gene profile, alginate production and biofilm formation in Iranian cystic fibrosis Pseudomonas aeruginosa isolates. Infez Med. 2018;26(3):226 236. 30246765

